# Factors associated with intent to stay in the profession: an exploratory cluster analysis across healthcare professions in Switzerland

**DOI:** 10.1093/eurpub/ckae100

**Published:** 2024-06-21

**Authors:** Leonard Roth, Ingrid Gilles, Emilie Antille, Jonathan Jubin, Vladimir Jolidon, Annie Oulevey-Bachmann, Isabelle Peytremann-Bridevaux

**Affiliations:** Department of Epidemiology and Health Systems, Centre for Primary Care and Public Health (Unisanté), University of Lausanne, Lausanne, Switzerland; Human Resources Direction, Lausanne University Hospital, Lausanne, Switzerland; Department of Epidemiology and Health Systems, Centre for Primary Care and Public Health (Unisanté), University of Lausanne, Lausanne, Switzerland; Department of Epidemiology and Health Systems, Centre for Primary Care and Public Health (Unisanté), University of Lausanne, Lausanne, Switzerland; La Source School of Nursing, HES-SO University of Applied Sciences and Arts Western Switzerland, Lausanne, Switzerland; Department of Epidemiology and Health Systems, Centre for Primary Care and Public Health (Unisanté), University of Lausanne, Lausanne, Switzerland; Department of Epidemiology and Health Systems, Centre for Primary Care and Public Health (Unisanté), University of Lausanne, Lausanne, Switzerland; La Source School of Nursing, HES-SO University of Applied Sciences and Arts Western Switzerland, Lausanne, Switzerland; Department of Epidemiology and Health Systems, Centre for Primary Care and Public Health (Unisanté), University of Lausanne, Lausanne, Switzerland

## Abstract

Retention issues are widespread within the health workforce. This cross-sectional study used data collected from 1707 healthcare professionals in 2022–23 to identify with *k*-means clustering groups of individuals sharing similar working experiences. These profiles were linked with varying levels of turnover intentions and a range of healthcare professions. While occupational therapists and paramedics reported in average better working conditions, registered nurses and intermediate caregivers reported the poorest experiences. In other clusters, salaries were high where work–life balance was low, and inversely. By learning from similarities and differences in the working conditions of diverse healthcare professionals, shared initiatives aimed at improving retention across professions can be facilitated.

## Introduction

Health systems face several health workforce crises: “a labor, a mental health, an education, a gender equality and a financial investment crisis” [1]. Affecting all health and care professions, the WHO Europe calls for action *now*, proposing to target the following five pillars: “Retain and Recruit”; “Optimize Performance”; “Build supply”; “Plan”; “Invest” [2]. To succeed in this task, precisely understanding the reasons why healthcare professionals (HCPs) leave their profession is crucial.

Since HCPs’ retention is key, many studies have sought to identify the factors associated with turnover intentions [3]. Although most often studied within single professions, it appears that several of these factors are common across healthcare professions, such as workload, work–life balance, remuneration, recognition, as well as professional development and career opportunities [4, 5].

Even though many general issues affecting the health workforce are similar across different professions, HCPs do not share identical working conditions and experiences, as highlighted by a recent study comparing perspectives from nurses and physicians in a hospital context [6]. Thus, while some professions exhibit certain similarities (i.e. the same set of associated factors), others may enjoy better working conditions and experiences in comparison.

Previous studies have mostly focused on nurses and physicians, within a limited scope of settings, so adopting a more multi-level and multi-professional approach is a priority goal for health services research [7]. Indeed, a better understanding of how the determinants of retention cluster across healthcare professions could facilitate the development and implementation of joint actions, thereby amplifying the impact of proposed solutions. Pooling knowledge and experiences can also help address multifaceted problems more effectively, fostering a supportive environment that would benefit groups of HCPs rather than single professions only.

With that in mind, this study aimed to identify clusters of HCPs with distinct patterns of working conditions and experiences, and to link these clusters with specific healthcare professions in the Swiss context.

## Methods

We conducted cross-sectional analyses of the first baseline survey of the Swiss COhort of Healthcare Professionals and Informal CAregivers (SCOHPICA, www.scohpica.ch). Between 1 October 2022 and 31 January 2023, any HCP practicing in Switzerland, irrespective of its employment status and work setting, was eligible to fill in the online questionnaire, which comprised socio-demographic, socio-professional, intention to leave/stay and wellbeing questions, as well as the determinants of the latter two. Ethical approval was obtained from the Cantonal Research Ethics Committee, Vaud (CER-VD), Switzerland (project ID: 2022-01410) and the project was registered on ClinicalTrials.gov, identifier: NCT05571488.

First, we derived a core set of factors associated with the intent to stay across healthcare professions (as this analysis was auxiliary to the main analysis, details are shown in the Supplementary material). Then, we ran a *k*-means clustering algorithm on the selected factors, which implied partitioning the multi-dimensional data points into *k* groups such that the squared Euclidean distances from points to the assigned cluster centers was minimized [8]. Thus, each cluster corresponded to a group of participants sharing similar working conditions and experiences. The optimal number of clusters was determined based on the strength of association with the intent to stay. Specifically, it was assessed trough the Bayesian Information Criterion [9], which selected the clustering solution that explained the greatest amount of variation in the intent to stay variable while using the fewest possible clusters. Finally, we described the clusters thus obtained through their centers (average values in each factor) and the proportion of HCPs from different professions they entailed. As the number of individuals varied across healthcare professions, the proportions were weighted so that the professional groups could be compared.

## Results

Data from 1707 HCPs were collected, out of which 33 were excluded because of incomplete information. Mean age was 42.5 years old (standard deviation: 11.6 years), 78% were women, 46% worked fulltime, and 54% had children. The 10 most represented professions were registered nurses (32.4%), physicians (12.4%), physiotherapists (9.1%), occupational therapists (5.3%), medical assistants (4.4%), pharmacists (4.2%), paramedics (3.6%), advanced practice nurses (3.6%), dietitians (3.4%) and intermediate caregivers (3.2%). While one third practiced in public hospitals, 27%, 9%, 12% worked in private practices, nursing homes and home care institutions, respectively. The determinants of the intent to stay measured in the survey are presented in Supplementary Table S1. The following seven were strongly associated in all settings: work-life balance, opportunities for development, meaning of work, workload, recognition, salary, and influence at work.

The optimal clustering solution based on the association with intent to stay in the profession identified five clusters (Supplementary Fig. S3). Table 1 presents a summary of the results, ranging from a cluster with high scores in most factors and the highest intent to stay, containing a relatively high proportion of occupational therapists and paramedics, to a cluster with low scores in most factors and the lowest intent to stay, containing a relatively high proportion of registered nurses and intermediate caregivers. While doctors and pharmacists were primarily grouped in a cluster characterized by high salaries and a poor work-life balance, dieticians and medical assistants stood out by reporting a good work-life balance but rather low salaries. Finally, physiotherapists and advanced practice nurses were linked with the cluster characterized by a moderate intent to stay in the profession and high meaning of work. The average scores for each cluster and each factor as well as the standardized proportions of HCPs assigned to the different clusters are shown in the Supplementary Fig. S4 and Supplementary Table S5.

**Table 1.** Intent to stay in the profession and semi-quantitative evaluation of associated factors, for each of the five clusters.

		Highest intent to stay and high (positive) scores	Moderately high intent to stay, high salary but poor work-life balance	Moderately high intent to stay, good work-life balance but low salary	Moderately low intent to stay but high meaning of work	Lowest intent to stay and low (negative) scores
		(*n* = 413)	(*n* = 260)	(*n* = 330)	(*n* = 450)	(*n* = 221)
	**Two most central professions in the cluster**	Occup. therapist Paramedic	PhysicianPharmacist	DietitianMedical assistant	Adv. practice nursePhysiotherapist	Registered nurse Int. caregiver
**Intent to stay (cluster’s mean score)**		**4.6**	**4.1**	**4.1**	**3.6**	**2.8**
**Factors associated with the intent to stay in the profession**	Work–life balance	**+**	**- -**	**++**	**-**	**-**
	Development possibilities	**++**	**+**	**-**	+-	**- -**
	Meaning of work	**++**	**+**	**-**	**++**	**- -**
	Workload	**++**	**-**	**++**	**- -**	**- -**
	Recognition	**++**	+/-	+/-	**-**	**- -**
	Salary	+/-	**++**	**- -**	**-**	**-**
	Influence at work	**++**	**+**	+/-	**-**	**- -**

*Note:* + and - are based on the average scores for participants in the corresponding cluster. Intent to stay in the profession was measured on a scale from 1 to 5, with 1 corresponding to *No, not at all* and 5 to *Yes, absolutely*. The two most central professions in each cluster correspond to the two professions with the highest weighted proportions in the cluster.

## Discussion

These results hint at patterns of working conditions and experiences more prevalent in certain professions. Negative patterns are apparent for intermediate caregivers and registered nurses, which can be mended by initiatives aimed at supporting them to find higher meaning in their work. Indeed, the ability to foster quality relationships with patients and family is often at the core of caregivers’ professional identity, and a lack thereof due to organizational deficiencies can have devastating consequences on the workforce [10]. On another hand, while the demand for a pay rise was emphasized for medical assistants and dietitians, this should preferably come without increased burden or workload, which could result in a deteriorated work-life balance.

Results from this study should be interpreted considering the following limitations. First, there is no nationwide registry of HCPs in Switzerland, so it was not feasible to use probability sampling and draw fully representative samples. However, socio-demographic characteristics of the respondents were compared with available statistics to ensure that the recruitment was comprehensive enough. Second, selection bias may naturally occur if individuals who chose not to respond differ significantly from survey participants. Without data on non-respondents, we were not able to assess the extent of this bias. Third, our research relied on self-reported data, which may be prone to recall and social desirability biases. To mitigate this, the SCOHPICA survey was designed based on widely used and validated questions and instruments. Fourth, the methods applied were exploratory, and there is a risk with clustering that a spurious partition is imposed on the data. In our case, while the clusters corresponded to distinct profiles of working conditions and experiences, the separation by profession was not always pronounced. Nevertheless, the patterns identified provide interesting insights and offer avenues for future analyses.

Further research around this project will involve validating the results by using both a newly recruited sample of HCPs and confirmatory statistical methods, and by organizing a stakeholder dialogue and a participants’ forum to discuss the findings’ implications.

To our knowledge, these cluster analyses are the first to have included multiple healthcare professions. They show promising results that need to be confirmed before enabling further transversal learning and considering joints actions. In the face of common challenges, collective approaches should ensure comprehensive solutions to improve working conditions, recruit and retain HCPs in the profession, and, ultimately, enhance the quality of patients care.

## Supplementary Material

ckae100_Supplementary_Data

## Data Availability

A Data Transfer Agreement is being implemented to grant the access to the study data upon any reasonable request. Please contact the corresponding author (leonard.roth@unisante.ch) or visit the SCOHPICA website (www.scohpica.ch) for further information. Key points A cluster analysis conducted on the working conditions and experiences of diverse healthcare professionals revealed five distinct patterns, each associated with varying levels of intent to stay in the profession. While certain healthcare professions, such as registered nurses and intermediate caregivers, were grouped together in the clusters reporting the most adverse working conditions, other professions distinguished themselves by exhibiting more positive patterns. The collective approaches and joint actions needed to solve the current health workforce crisis should be informed by both common aspects and important disparities in the working life of healthcare professionals across various professions.
