# Supplementary material for: Factors associated with intent to stay in the profession: an exploratory cluster analysis across healthcare professions in Switzerland
Source: Eur J Public Health. 2024 Jun 21;34(6):1146–8. doi: 10.1093/eurpub/ckae100 (PMC11631472; doi:10.1093/eurpub/ckae100)
Supplement: ckae100_Supplementary_Data [file ckae100_supplementary_data.pdf]

Supplementary Material

Data from the baseline SCOHPICA survey was used to derive a core set of factors associated with the intent to stay across healthcare professions. Intent to stay in the profession was assessed through the question “If your working conditions/environment were to remain the same over the next few months, would you stay in your current profession?” and responses were measured on a 5-point scale, ranging from “No, not at all” to “Yes, absolutely”. The list of determinants of the intent to stay measured in the survey as well as their origins and definitions are presented in Table S1 below. They were all standardised before being used in the model. Higher scores indicated better working conditions and experiences. Socio-demographic characteristics and socio-professional information, in particular age, gender, education, employment status, activity rate, work location and years in the profession, were treated as potential confounders. Complete data was obtained for 1’674 participants.

The regression framework was a multivariable linear regression. A core set of factors was identified with backward stepwise variable selection based on likelihood ratio tests for the coefficients. Thus, a model was constructed that explained as much of the outcome as possible while favouring parsimony. The robustness of the analysis was checked with an ordinal logistic regression, which did not alter the results in any meaningful way.

Figure S2 below shows the parameter values estimated with the final model. After adjustment for other covariates, work-life balance had the largest effect on intent to stay in this data, followed by opportunities for development, meaning of work, workload, recognition, salary, and influence at work. All associations had p-values < 0.001.

Supplementary Table S1: Determinants of the intent to stay in the profession measured in the baseline SCOHPICA survey.

Dimension	Instrument	Description
Workload	Quantitative Workload Inventory	Perceived amount of work in terms of pace and volume
Staffing & resources	Practice Environment Scale of the Nursing Work Index	Staffing and resource adequacy to work
Opportunities for development	Copenhagen Psychosocial Questionnaire	Possibility to learn new things at work, to use skills/expertise, to develop competences
Work-life balance	Copenhagen Psychosocial Questionnaire	Interference of work demands with private life, work drains energy and has negative effects on private life
Work readiness	NA	Feeling prepared for professional activity by previous training
Recognition at work	Recognition at Work Scale	Recognition by the company, colleagues, and managers
Meaning of work	Copenhagen Psychosocial Questionnaire	Feeling that the work done is meaningful and important
Leadership	Global Transformational Leadership Scale	Providing a vision and an appropriate model, fostering the acceptance of group goals, performance expectations, providing individualized support to staff and intellectual stimulation
Control over working time	Copenhagen Psychosocial Questionnaire	Ability to decide when to take holiday; control over work overtime
Influence at work	Copenhagen Psychosocial Questionnaire	Degree of influence on the decisions at work
Sense of community at work	Copenhagen Psychosocial Questionnaire	Atmosphere and co-operation with colleagues
Interprofessional collaboration	Intensity of Interprofessional Collaboration	Collaboration between interprofessional team members
Moral resilience	Rushton Moral Resilience Scale	The capacity to sustain/restore integrity in response to moral adversity
Salary	Six categories based on official national statistics	Personal income from employment

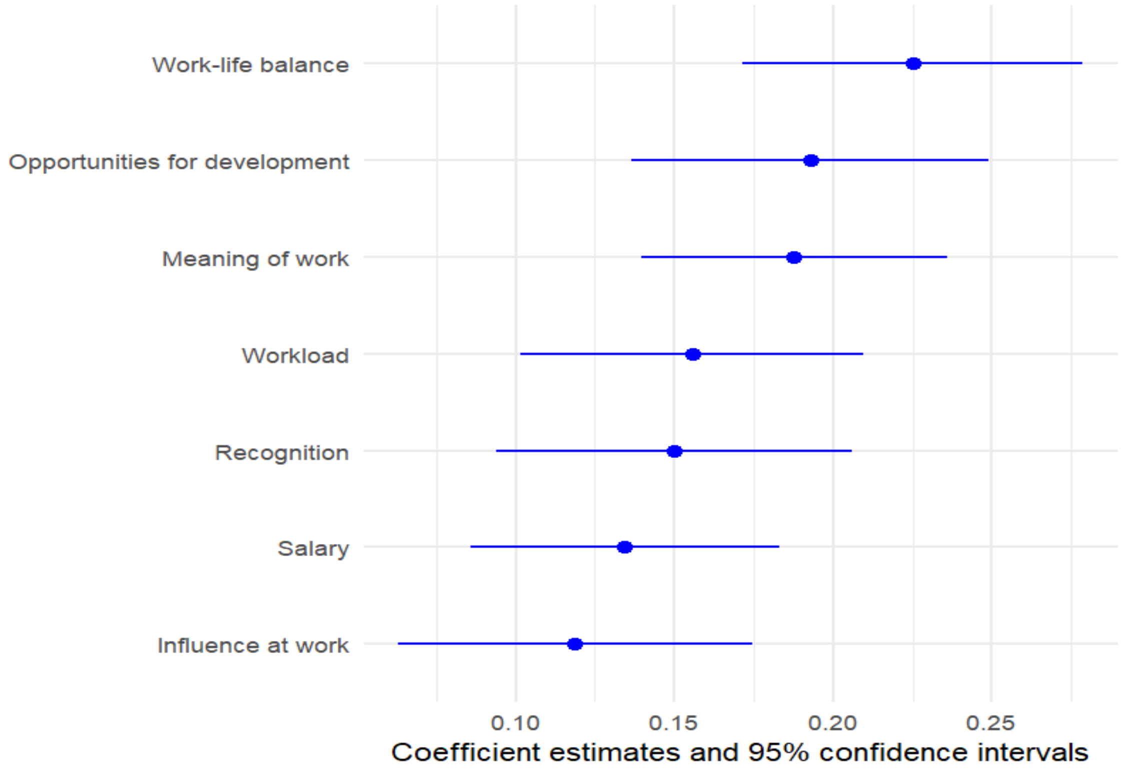

Supplementary Figure S2: Core factors associated with the intent to stay in the profession derived with a multivariable regression model (n = 1'674).

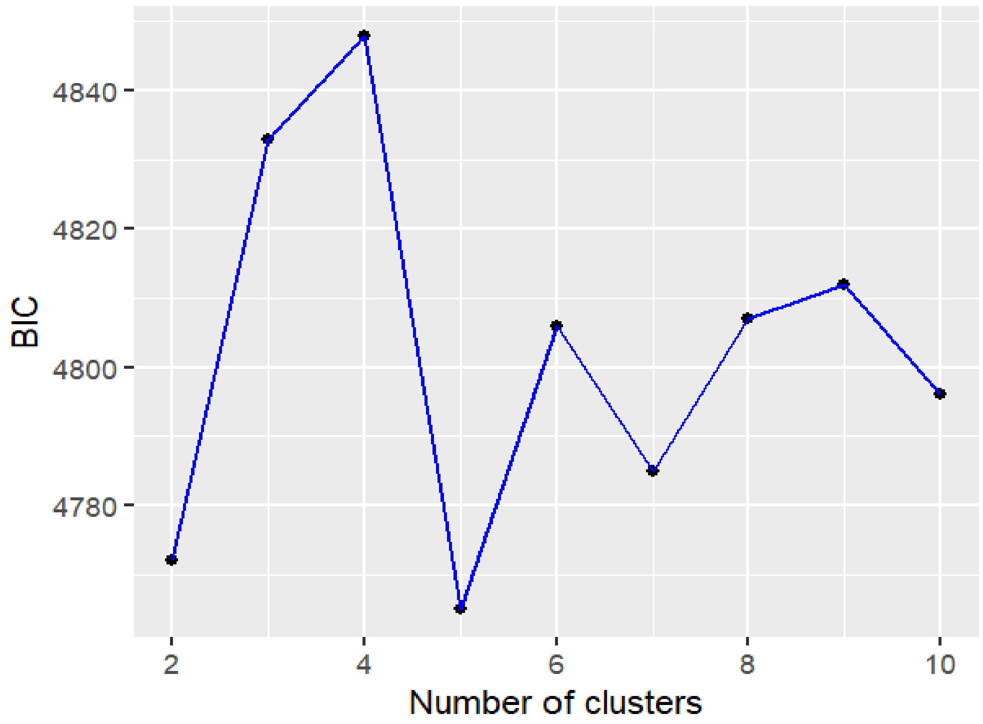

Supplementary Figure S3: Bayesian Information Criterion (BIC) corresponding to the regression models with intent to stay in the profession as independent variable and cluster membership as dependent variable, for varying numbers of clusters (n = 1'674). The model with the lowest BIC is chosen (five clusters) as it explains the most variation in the independent variable while penalizing complexity in the dependent variable.

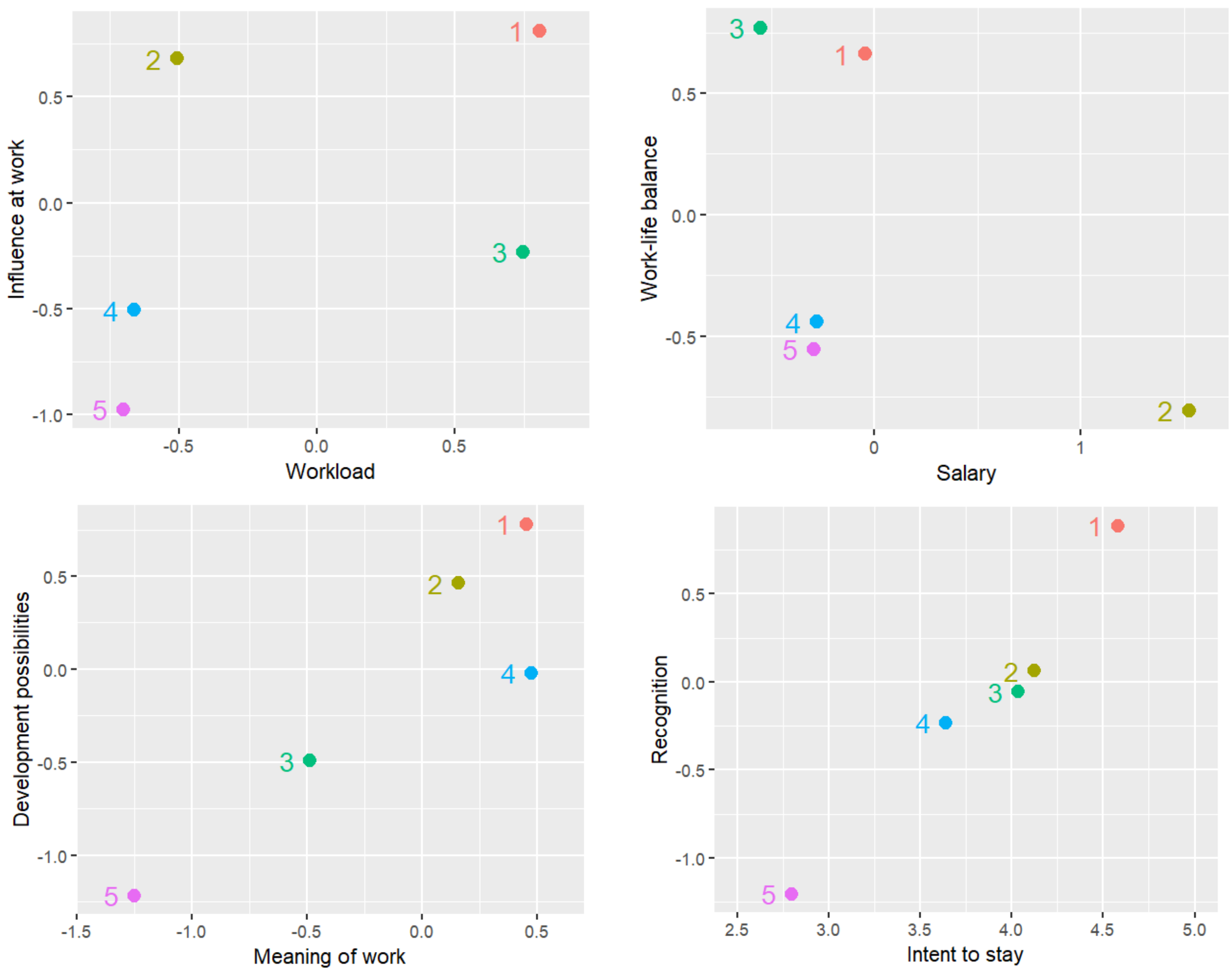

Supplementary Figure S4: Average scores in the determinants of the intent to stay in the profession, by cluster. The intent itself (bottom right) was not included in the clustering procedure but was used to select the optimal number of clusters (see Figure S3). Higher scores indicate more positive working conditions and experiences. Cluster 1 represents the group with highest intent to stay and high scores generally, cluster 2 represents the group with moderately high intent to stay, high salary but poor work-life balance, cluster 3 represents the group with moderately high intent to stay, good work-life balance but low salary, cluster 4 represents the group with moderately low intent to stay but high meaning of work, and cluster 5 represents the group with lowest intent to stay and low scores generally. We see that cluster 1 is distinct from the other clusters in all dimensions except work-life balance, meaning of work and salary. Cluster 2 and 3 are at times close to each other but at opposite end of the spectrum in terms of work-life balance and salary (top right). Cluster 4 and 5 are relatively similar except for development possibilities and meaning of work (bottom left).

Supplementary Table S5: Relative proportions of individuals from the healthcare professions with n > 50 in each cluster. The proportions were standardized (weighted) based on the total proportions for each profession in our study sample. The rows highlighted correspond to the two most “central” professions in each cluster. In some cases, the separation is clear (second cluster for instance); in some cases, less so (third cluster for instance).

Highest intent to stay and high (positive) scores (n=413)		*Moderately high intent to stay, high salary but poor work-life balance (n=260)*		*Moderately high intent to stay, good work-life balance but low salary (n=330)*		*Moderately low intent to stay but high meaning of work (n=450)*		*Lowest intent to stay and low (negative) scores (n=221)*	
Occup. Therapist	16.7%	Physician	39.3%	Dietitian	18.9%	Adv. Practice Nurse	16.8%	Registered Nurse	19.7%
Paramedic	16.1%	Pharmacist	21.8%	Medical Assistant	14.1%	Physio-therapist	15.6%	Int. Caregiver	17.9%
Physio-therapist	13.2%	Adv. Practice Nurse	15.6%	Paramedic	13.7%	Registered Nurse	11.9%	Medical Assistant	13.2%
Medical Assistant	12.9%	Physio-therapist	7.5%	Int. Caregiver	11.9%	Int. Caregiver	11.3%	Pharmacist	11.6%
Dietitian	9.4%	Paramedic	5.3%	Occup. Therapist	11.9%	Pharmacist	11.1%	Adv. Practice Nurse	10%
Adv. Practice Nurse	7.7%	Registered Nurse	3.7%	Physio-therapist	9%	Medical Assistant	8%	Physician	7.7%
Registered Nurse	6.9%	Dietitian	3.5%	Registered Nurse	7.2%	Occup. Therapist	7.8%	Dietitian	7.3%
Pharmacist	6.3%	Occup. Therapist	2.2%	Adv. Practice Nurse	7.1%	Dietitian	7%	Physio-therapist	4.8%
Physician	5.5%	Int. Caregiver	1.2%	Pharmacist	4.6%	Paramedic	5.3%	Occup. Therapist	4.5%
Int. Caregiver	5.4%	Medical Assistant	0%	Physician	1.7%	Physician	5.3%	Paramedic	3.4%
